# Neonatal corticosterone administration increases p27-positive Sertoli cell number and decreases Sertoli cell number in the testes of mice at prepuberty

**DOI:** 10.1038/s41598-022-23695-8

**Published:** 2022-11-12

**Authors:** Hidenobu Miyaso, Kaiya Takano, Kenta Nagahori, Zhong-Lian Li, Shinichi Kawata, Miyuki Kuramasu, Yuki Ogawa, Hirotaka Yoshioka, Yoshiharu Matsuno, Satoshi Yokota, Masahiro Itoh

**Affiliations:** 1 Department of Anatomy, Faculty of Medicine, School of Medicine, International University of Health and Welfare, 4-3 Kozunomori, Narita, Chiba 286-8686 Japan; 2 Department of Anatomy, Tokyo Medical University, 6-1-1 Shinjuku, Shinjuku-Ku, Tokyo, 160-8402 Japan; 3 Center for Basic Medical Research, Narita Campus, International University of Health and Welfare, 4-3 Kozunomori, Narita, Chiba 286-8686 Japan; 4 Division of Cellular & Molecular Toxicology, Center for Biological Safety & Research, National Institute of Health Sciences, 3-25-26 Tono-Machi, Kawasaki-Ku, Kawasaki, Kanagawa 210-9501 Japan

**Keywords:** Developmental biology, Environmental sciences, Endocrinology, Health care, Risk factors

## Abstract

Cortisol and corticosterone (CORT) are steroid, antistress hormones and one of the glucocorticoids in humans and animals, respectively. This study evaluated the effects of CORT administration on the male reproductive system in early life stages. CORT was subcutaneously injected at 0.36 (low-), 3.6 (middle-), and 36 (high-dosed) mg/kg body weight from postnatal day (PND) 1 to 10 in ICR mice. We observed a dose-dependent increase in serum CORT levels on PND 10, and serum testosterone levels were significantly increased only in high-dosed-CORT mice. Triiodothyronine levels were significantly higher in the low-dosed mice but lower in the middle- and high-dosed mice. However, testicular weights did not change significantly among the mice. Sertoli cell numbers were significantly reduced in low- and middle-dosed mice, whereas p27-positive Sertoli cell numbers increased in low- and middle-dosed mice. On PND 16, significant increases in testicular and relative testicular weights were observed in all-dosed-CORT mice. On PND 70, a significant decrease in testicular weight, Sertoli cell number, and spermatozoa count was observed. These results revealed that increased serum CORT levels in early life stages could induce p27 expression in Sertoli cells and terminate Sertoli cell proliferation, leading to decreased Sertoli cell number in mouse testes.

## Introduction

Cortisol and corticosterone (CORT) are steroid, antistress hormones and one of the glucocorticoids in humans and animals, respectively. CORT is secreted through the hypothalamus–pituitary–adrenal (HPA) axis and is involved in energy regulation^1,2^. They are secreted following exposure to stress and assist humans and animals cope with several stressful situations^3^. Notably, several studies have demonstrated that excessive secretion of CORT following exposure to extreme mental or physical stresses in early life stages, i.e., early life stress (ELS), has adverse effects on the health of humans and animals in later life^4–11^. In addition, it has been reported that glucocorticoid administration during early life stages causes a decrease in brain weight, reduction in myelination of fibers, persistent downregulation of glucocorticoid receptors (GRs), impairment of adrenocortical response to stress, learning disturbance, developmental delay, change in HPA reactivity, and increase in depressive-like behavior^12–21^. Therefore, long-term and extreme elevation of glucocorticoid levels with chronic external and environmental stresses in early life may be neurotoxic and affect the nervous system^20^. In contrast, increased testicular weight and 3β-hydroxysteroid dehydrogenase activity as well as advanced testis descent at puberty were observed in the male reproductive system following neonatal CORT administration^22^. Nevertheless, the toxicological mechanisms underlying this phenomenon and other toxicities induced in the male reproductive system as a result of CORT administration remain unclear.

Neonatal maternal separation (NMS) is a representative model for inducing ELS. NMS decreased testicular weight in the male reproductive system of mice^23,24^. Furthermore, NMS reduced the numbers of epididymal spermatozoa and Sertoli cells on postnatal day (PND) 70^25^. Moreover, a previous study demonstrated that NMS decreased the Sertoli cell number on PND 16 and increased the p27-positive Sertoli cell number on PND 10^26^. P27 is a cyclin-dependent kinase (CDK) inhibitor, and elevated p27 expression levels in Sertoli cells have been reported to terminate Sertoli cell proliferation^27^. However, the mechanisms by which NMS increases the number of p27-positive Sertoli cells, thereby decreasing the Sertoli cell number, remain unclear.

It has been reported that several hormones regulate p27 induction. Buzzard et al. showed that testosterone and triiodothyronine (T3) treatments induced p27 expression in cultured rat Sertoli cells^28^. On the other hand, growing data indicate that NMS or ELS can change the levels of both testosterone and T3 at prepuberty and postpuberty. For example, Tsuda et al. reported that 3-h/day NMS during PND 1–14 decreased serum testosterone levels at 4, 5, and 6 weeks of age^23^. Jaimes-Hoy et al. also documented a decrease in serum thyroid hormone levels on PND 90 as a result of 3-h/day NMS during PND 2–21^29^. Our abovementioned experimental model, i.e., 2-h/day NMS during PND 1–10, demonstrated that serum testosterone levels significantly decreased in NMS mice on PND 10; however, T3 levels did not significantly alter^26^.

In a previous study, we also observed that serum CORT levels increased on PND 10 in NMS mice^26^. Studies have indicated that CORT decreases the level of testosterone, hormone that is important for increasing the Sertoli cell number^30,31^. Jiang et al. reported that CORT induced p27 expression in mouse mammary hyperplastic epithelial cell lines (TM-10 cells)^32^. Moreover, several studies have indicated the expression of GRs in Sertoli cells^33–35^. Thus, we hypothesized that an increase in the CORT level in NMS mice at prepuberty may trigger changes in the serum testosterone level or p27-positive Sertoli cell number and decrease the Sertoli cell number. In this study, we administered CORT to newborn mice to test our hypothesis and delineate a precise mechanism by which NMS decreases the Sertoli cell number. We evaluated the effects of CORT administration on the male reproductive system, particularly the Sertoli cell number, p27-positive Sertoli cell number, and several hormonal levels on PNDs 10, 16, and 70.

## Results

### Serum hormone levels in CORT mice on PNDs 10 and 16

**Figure 1:**
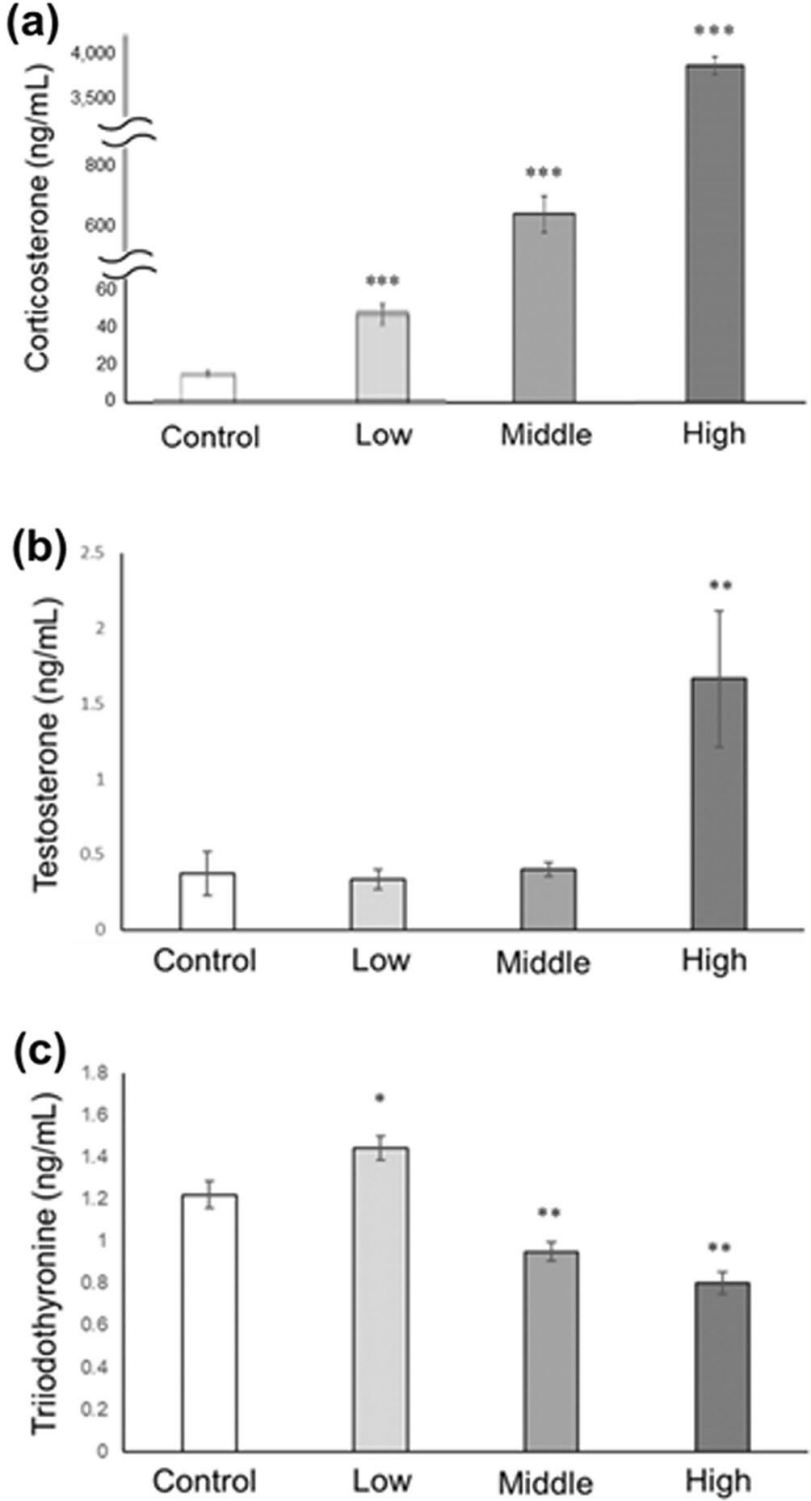
Serum CORT, testosterone, and T3 levels in CORT-administered mice on PND 10. Serum CORT (**a**), testosterone (**b**), and T3 (**c**) levels on PND 10 were assessed using ELISA. Values are expressed as the mean ± S.E.M of 12 samples per group. *p < 0.05, **p < 0.01, and ***p < 0.001.

**Figure 2:**
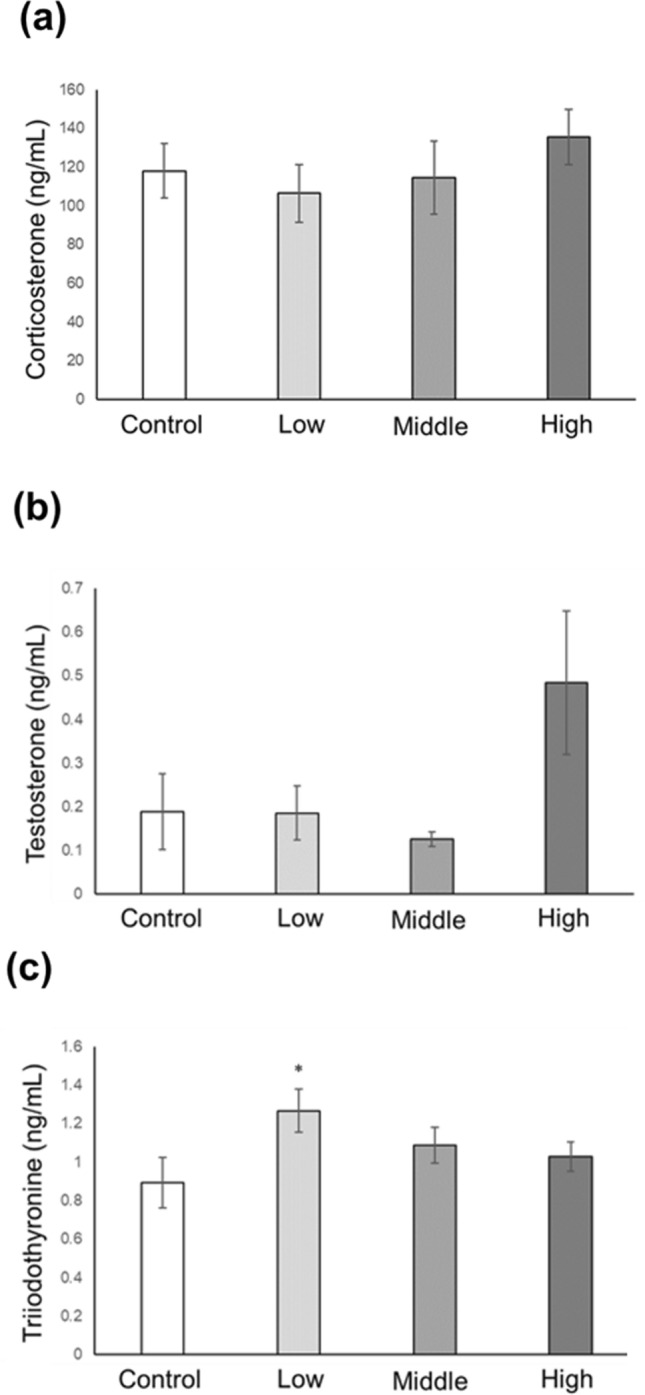
Serum CORT, testosterone, and T3 levels in CORT-administered mice on PND 16. Serum CORT (**a**), testosterone (**b**), and T3 (**c**) levels on PND 16 were assessed using ELISA. Values are expressed as the mean ± S.E.M of 12 samples per group. *p < 0.05.

On PND 10, serum CORT levels were found to be significantly increased in CORT mice compared with those in control mice in a dose-dependent manner (Fig. 1a). Serum testosterone levels significantly increased in high-dosed-CORT mice (Fig. 1b). T3 levels significantly increased in low-dosed-CORT mice and decreased in middle- and high-dosed-CORT mice (Fig. 1c). On PND 16, no significant differences in serum CORT and testosterone levels were observed between control and all dosed-CORT mice (Fig. 2a,b). However, serum T3 levels significantly increased in low-dosed-CORT mice compared with those in control mice (Fig. 2c).

### Body weight and testicular weight of CORT mice on PNDs 10 and 16

Compared with control mice, the body weight (BW) of low- and high-dosed-CORT mice significantly decreased on PND 10 (Table 1). However, no differences in the testicular weights were observed among control and all-dosed-CORT mice. The relative testicular weight (organ weight/BW × 100) of high-dosed-CORT mice had significantly increased. On PND 16, no differences in BW were observed among control and all dosed-CORT mice (Table 2). The testicular weight and relative testicular weight were higher in all dosed-CORT mice than those in control mice.

**Table 1:** Body and testicular weights of CORT-administered mice on PND 10.

	Control	Low dose	Middle dose	High dose
Body weight (g)	7.20 ± 0.210	6.49 ± 0.11*	6.88 ± 0.20	5.62 ± 0.10***
Testicular weight (g) × 100	0.559 ± 0.029	0.577 ± 0.028	0.604 ± 0.038	0.538 ± 0.019
Relative testicular weight (organ weight/body weight × 100)	7.764 ± 0.344	8.906 ± 0.442	8.773 ± 0.485	9.597 ± 0.325**

Values are expressed as the mean ± S.E.M. of 12 samples per group.

*p < 0.05, **p < 0.01, and ***p < 0.001.

*CORT* corticosterone.

**Table 2:** Body and testicular weights of CORT-administered mice on PND 16.

	Control	Low dose	Middle dose	High dose
Body weight (g)	8.78 ± 0.27	9.42 ± 0.21	8.83 ± 0.13	8.52 ± 0.10
Testicular weight (g) × 100	1.033 ± 0.045	1.289 ± 0.052*	1.383 ± 0.061**	1.325 ± 0.047**
Relative testicular weight (organ weight/body weight × 100)	11.800 ± 0.411	13.665 ± 0.390*	15.688 ± 0.691***	15.584 ± 0.592**

Values are expressed as the mean ± S.E.M. of 12 samples per group.

*p < 0.05, **p < 0.01, and ***p < 0.001.

*CORT* corticosterone.

### Testicular histology in CORT mice on PNDs 10 and 16

**Figure 3:**
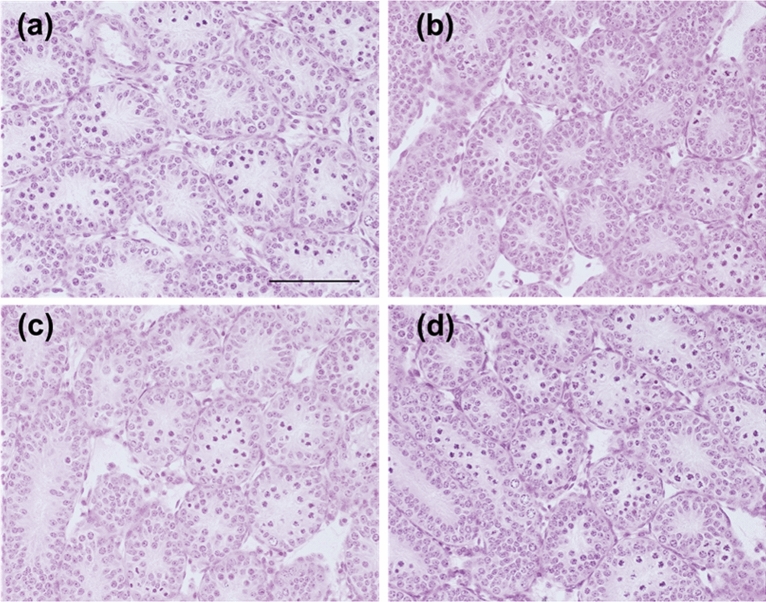
Testicular histology of CORT-administered mice on PND 10. Testicular histology of control (**a**), low-dosed (**b**), middle-dosed (**c**), and high-dosed-CORT mice (**d**) on PND 10. Representative hematoxylin and eosin (H&E)-stained micrographs are provided. A decrease in the diameter of seminiferous tubules and the height of the seminiferous epithelia were noted in the low- and high-dosed-CORT mice compared to the control. The scale bar is 100 µm.

**Figure 4:**
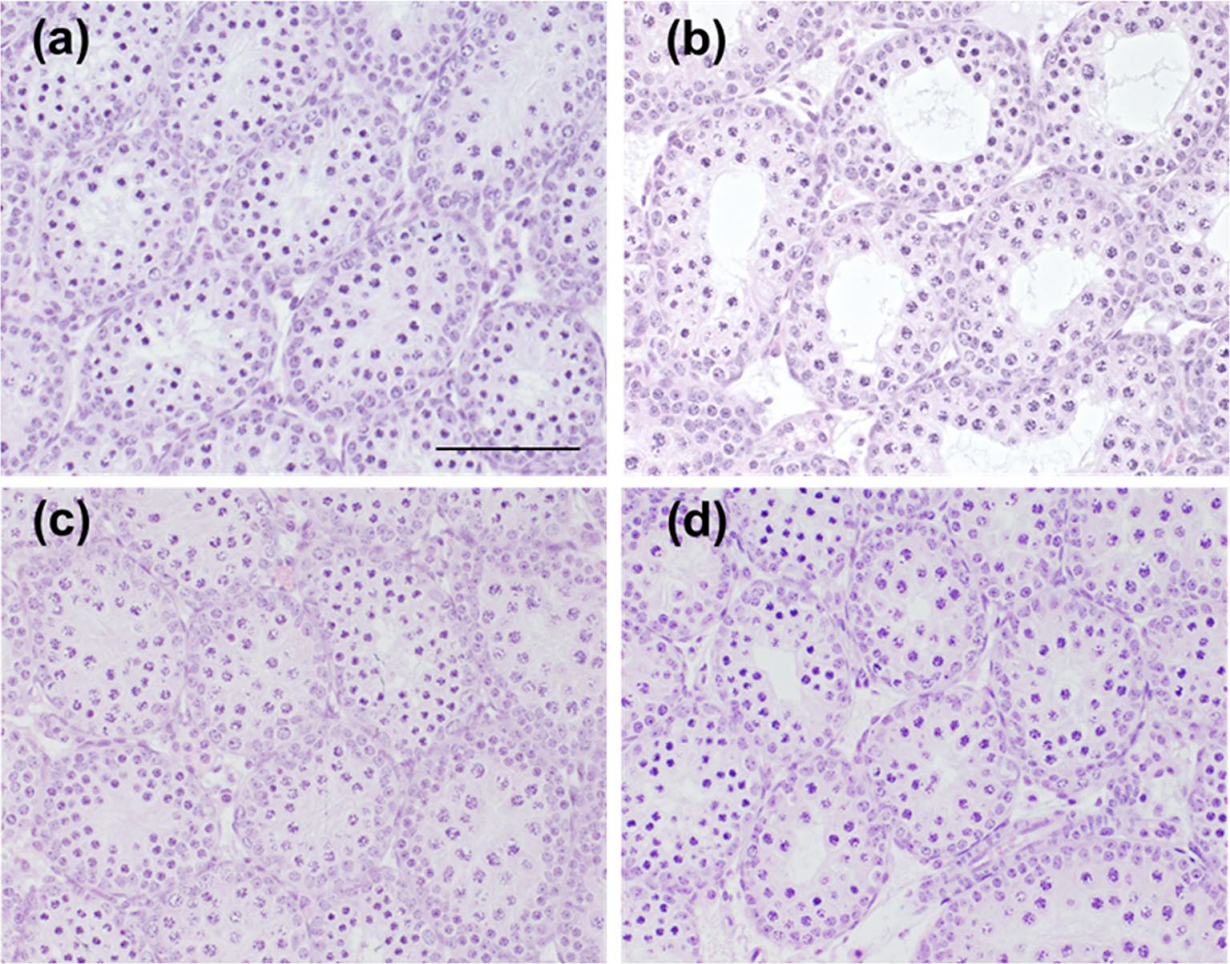
Testicular histology of CORT-administered mice on PND 16. Testicular histology of control (**a**), low-dosed (**b**), middle-dosed (**c**), and high-dosed-CORT mice (**d**) on PND 16. Representative H&E-stained micrographs are provided. The scale bar is 100 µm.

On PND 10, the diameters of the seminiferous tubules were significantly decreased in low- and high-dosed-CORT mice compared with those in control mice (Fig. 3a–d and Table 3). In middle-dosed-CORT mice, the height of the seminiferous epithelium significantly increased, whereas it considerably decreased in low- and high-dosed-CORT mice. Middle-dosed-CORT mice had a significantly lower relative interstitial area (interstitial tissue area/total testicular tissue area). Testicular histological evaluation on PND 16 revealed that the seminiferous tubule diameters were significantly decreased in high-dosed-CORT mice compared with those in control mice (Fig. 4a–d and Table 4). The seminiferous epithelium height significantly increased in middle-dosed-CORT mice but decreased in high-dosed-CORT mice. The relative interstitial area significantly decreased in all-dosed-CORT mice.

**Table 3:** Testicular morphology of CORT-administered mice on PND 10.

	Control	Low dose	Middle dose	High dose
Diameter of seminiferous tubule (µm)	91.76 ± 1.76	77.95 ± 1.55***	94.31 ± 1.61	79.81 ± 1.69***
Height of seminiferous epithelium (µm)	41.19 ± 0.74	35.49 ± 0.91***	44.23 ± 1.08**	36.2 ± 0.81***
Relative interstitial area (interstitial tissue area to the total testicular tissue area)	0.193 ± 0.006	0.209 ± 0.011	0.165 ± 0.005**	0.183 ± 0.013

Values are expressed as the mean ± S.E.M. of data from 10 animals per group (10 seminiferous tubules or pictures per animal).

**p < 0.01, and ***p < 0.001 versus the control group.

*CORT* corticosterone.

**Table 4:** Testicular morphology of CORT-administered in mice on PND 16.

	Control	Low dose	Middle dose	High dose
Diameter of seminiferous tubule (µm)	117.41 ± 1.94	121.18 ± 2.01	114.54 ± 2.30	105.91 ± 1.64***
Height of seminiferous epithelium (µm)	43.71 ± 1.02	42.2 ± 1.38	49.98 ± 1.20***	37.96 ± 1.00***
Relative interstitial area (interstitial tissue area to the total testicular tissue area)	0.195 ± 0.019	0.132 ± 0.005***	0.119 ± 0.006***	0.157 ± 0.012***

Values are expressed as the mean ± S.E.M. of data from 10 animals per group (10 seminiferous tubules or pictures per animal).

***p < 0.001 versus the control group.

*CORT* corticosterone.

### Sertoli cell number and p27-positive Sertoli cell number in CORT mice on PNDs 10 and 16

**Figure 5:**
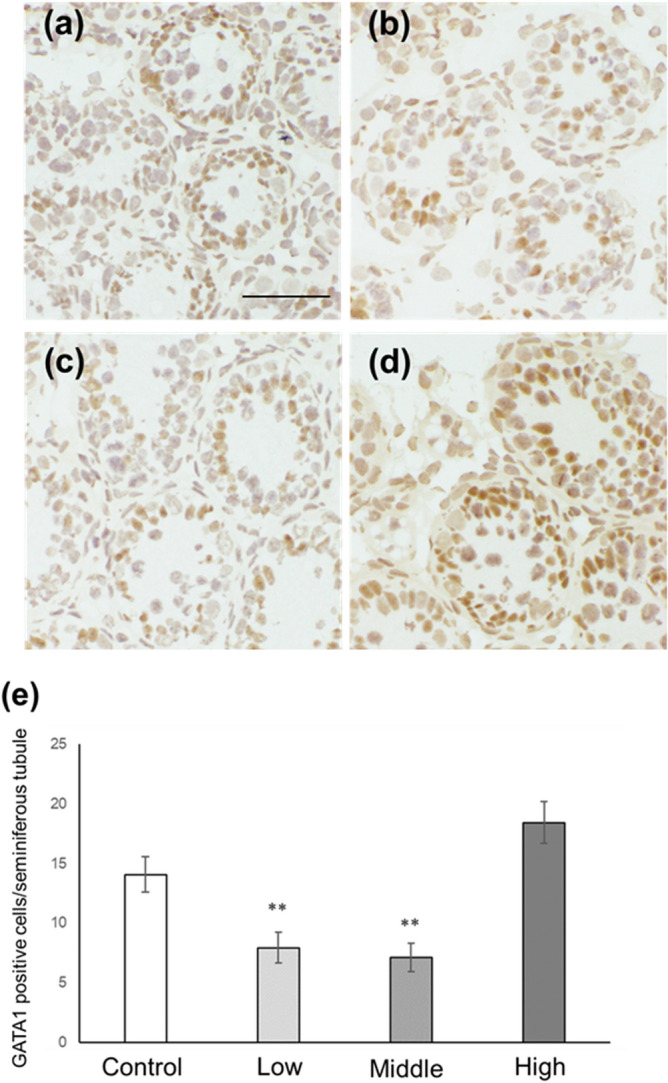
Number of GATA-1-positive Sertoli cells in CORT-administered mice on PND 10. Representative micrographs of Sertoli cells immunostained with anti-GATA-1 antibody in the testes from control (**a**), low-dosed (**b**), middle-dosed (**c**), and high-dosed-CORT mice (**d**) on PND 10. The scale bar is 50 µm. GATA-1-positive cells per seminiferous tubule among the control and CORT mice (**e**). Values are expressed as the mean ± S.E.M of data from 10 animals per group (10 seminiferous tubules per animal). **p < 0.01 compared to the control.

**Figure 6:**
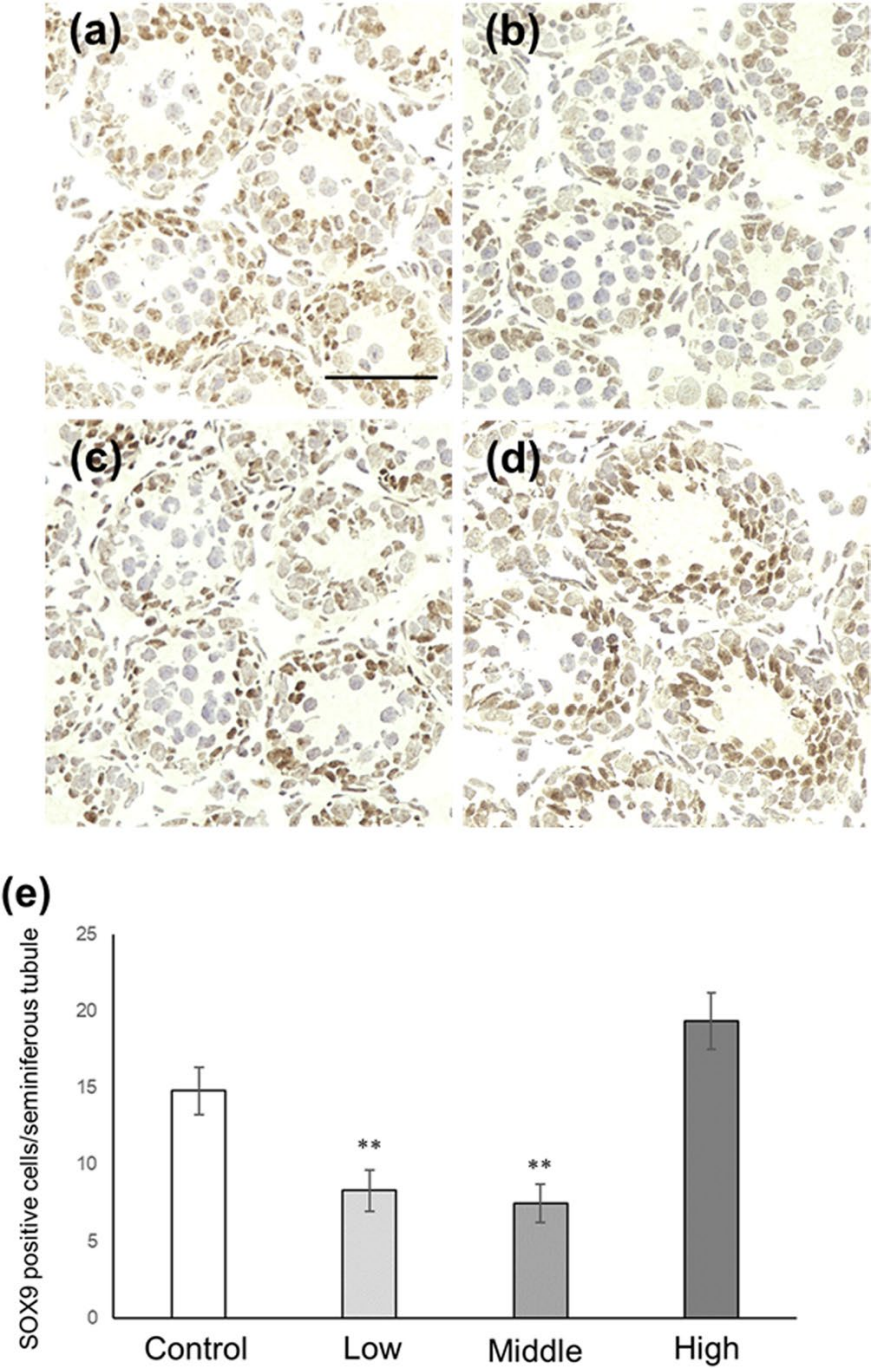
Number of SOX-9-positive Sertoli cells in CORT-administered mice on PND 10. Representative micrographs of Sertoli cells immunostained with anti-SOX-9 antibody in the testes from control (**a**), low-dosed (**b**), middle-dosed (**c**), and high-dosed-CORT mice (**d**) on PND 10. The scale bar is 50 µm. SOX-9-positive cells per seminiferous tubule among the control and CORT mice (**e**). Values are expressed as the mean ± S.E.M of data from 10 animals per group (10 seminiferous tubules per animal). **p < 0.01 compared to the control.

**Figure 7:**
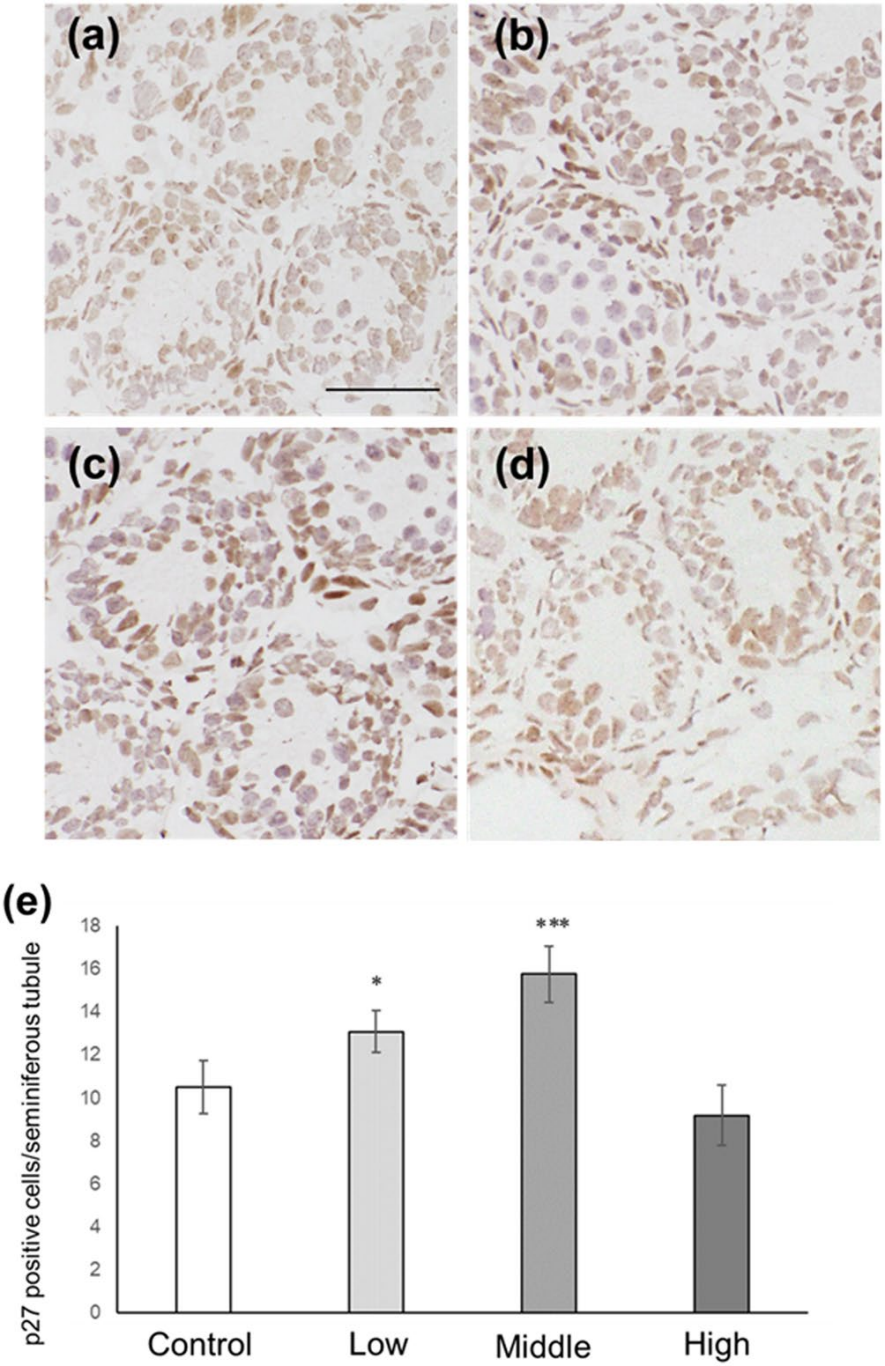
Number of p27-positive Sertoli cells in CORT-administered mice on PND 10. Representative micrographs of Sertoli cells immunostained with anti-p27 antibody in the testes from control (**a**), low-dosed (**b**), middle-dosed (**c**), and high-dosed-CORT mice (**d**) on PND 10. The scale bar is 50 µm. P27-positive cells per seminiferous tubule among the control and CORT mice (**e**). Values are expressed as the mean ± S.E.M of data from 10 animals per group (10 seminiferous tubules per animal). *p < 0.05 and ***p < 0.001 compared to the control.

**Figure 8:**
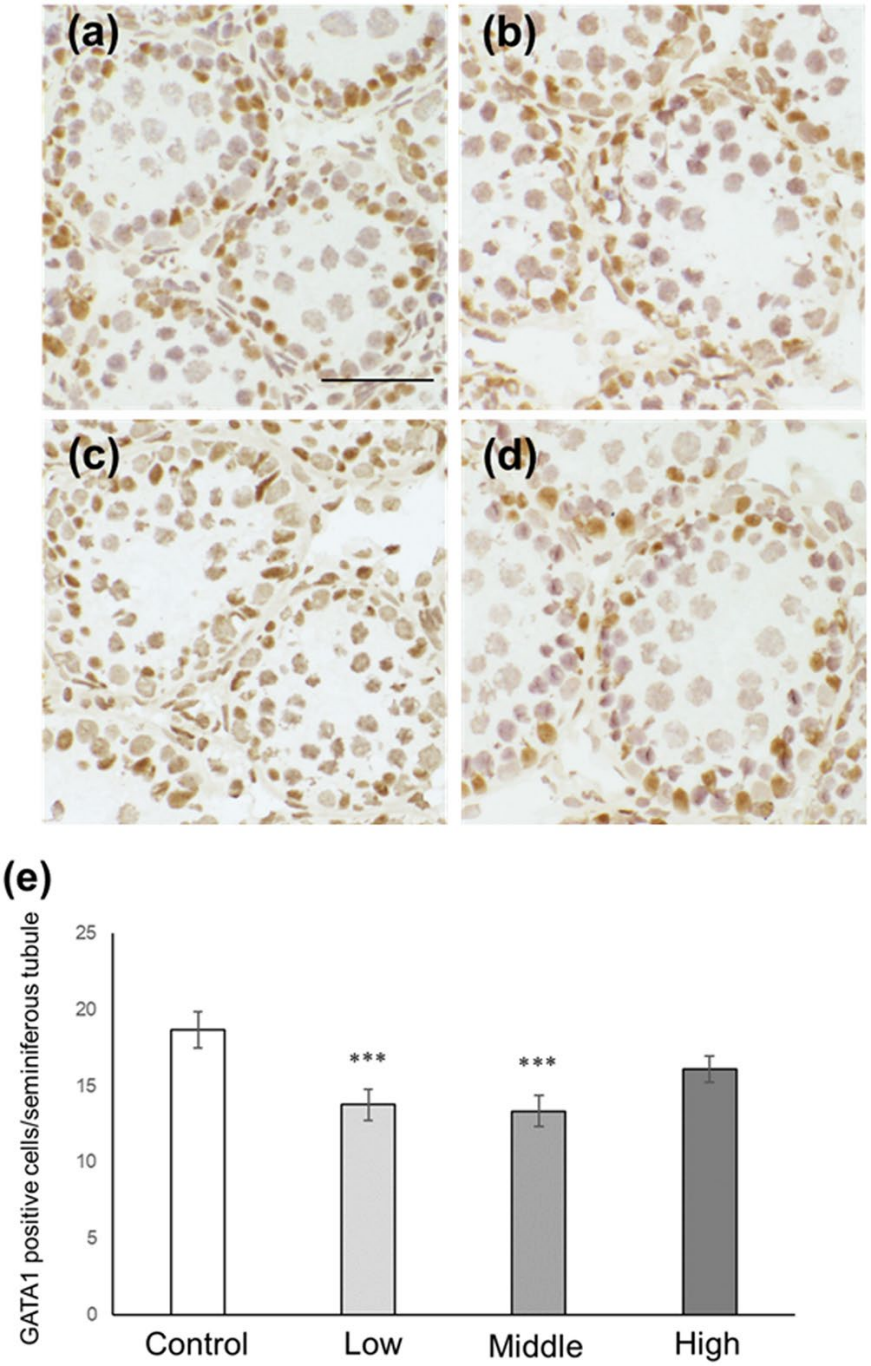
Number of GATA-1-positive Sertoli cells in CORT-administered mice on PND 16. Representative micrographs of Sertoli cells immunostained with anti-GATA-1 antibody in the testes from control (**a**), low-dosed (**b**), middle-dosed (**c**), and high-dosed-CORT mice (**d**) on PND 16. The scale bar is 50 µm. GATA-1-positive cells per seminiferous tubule among the control and CORT mice (**e**). Values are expressed as the mean ± S.E.M of data from 10 animals per group (10 seminiferous tubules per animal). ***p < 0.001 compared to the control.

**Figure 9:**
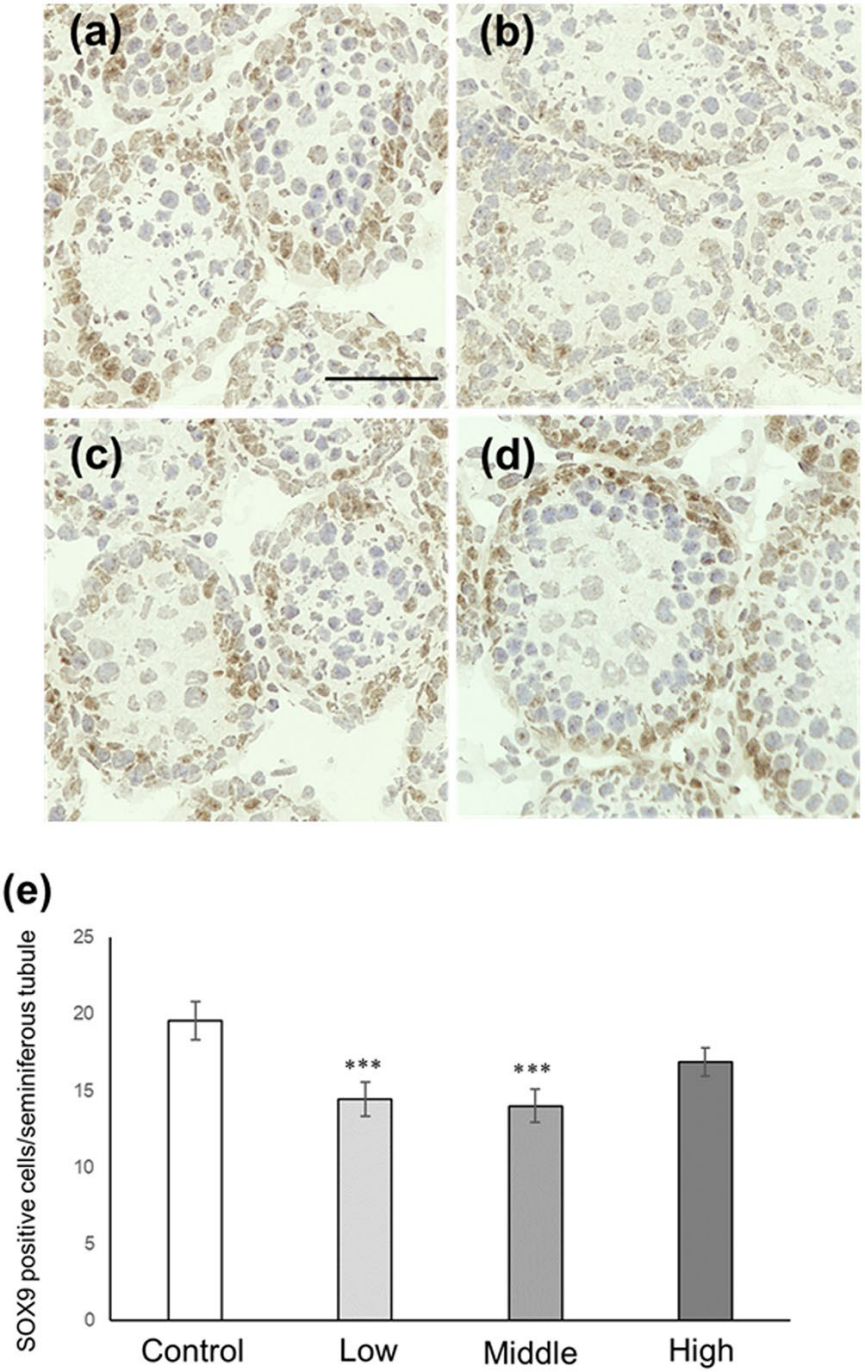
Number of SOX-9-positive Sertoli cells in CORT-administered mice on PND 16. Representative micrographs of Sertoli cells immunostained with anti-GATA-1 antibody in the testes from control (**a**), low-dosed (**b**), middle-dosed (**c**), and high-dosed-CORT mice (**d**) on PND 16. The scale bar is 50 µm. SOX-9-positive cells per seminiferous tubule among the control and CORT mice (**e**). Values are expressed as the mean ± S.E.M of data from 10 animals per group (10 seminiferous tubules per animal). ***p < 0.001 compared to the control.

**Figure 10:**
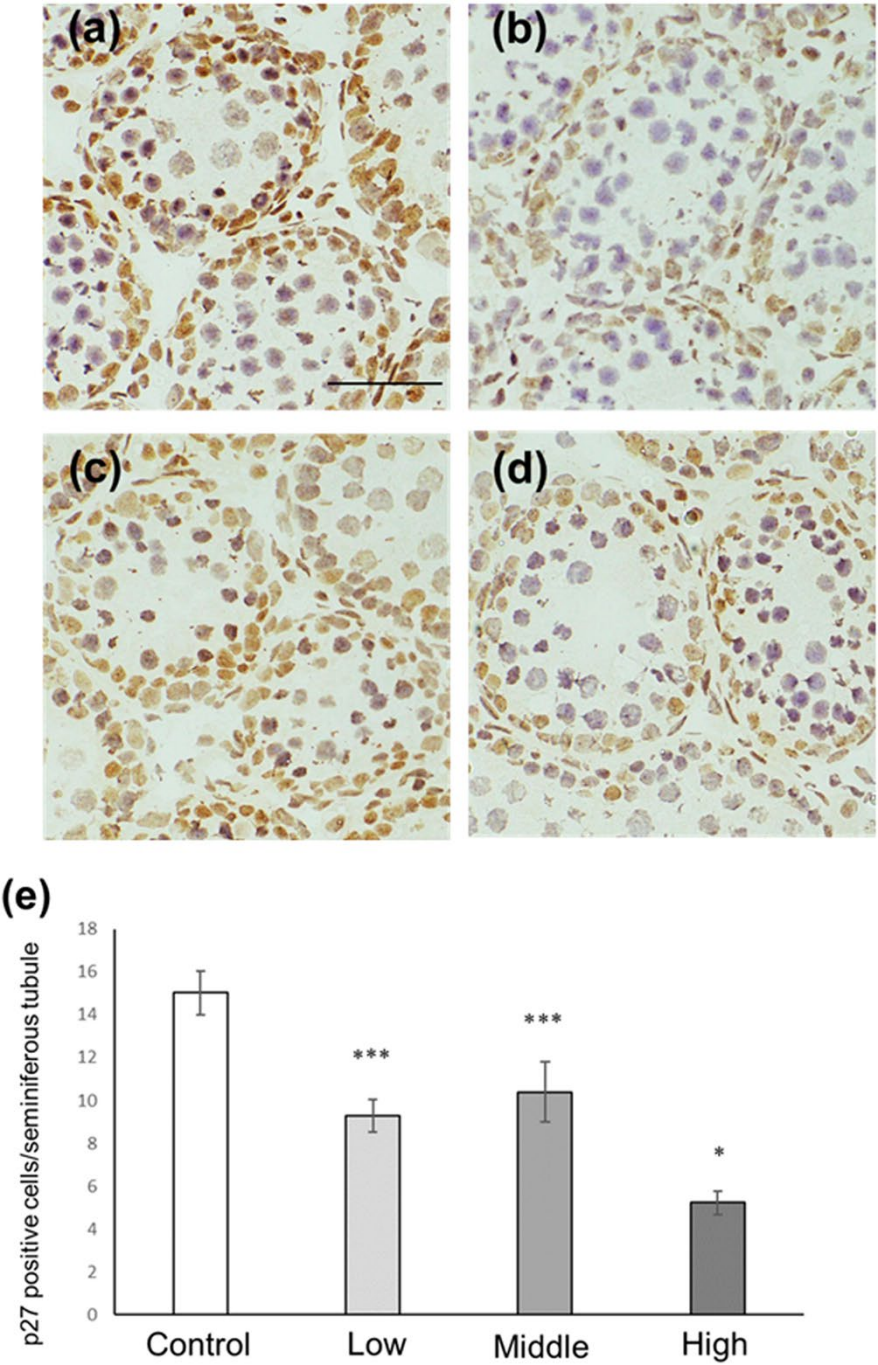
Number of p27-positive Sertoli cells in CORT-administered mice on PND 16. Representative micrographs of Sertoli cells immunostained with anti-p27 antibody in the testes from control (**a**), low-dosed (**b**), middle-dosed (**c**), and high-dosed-CORT mice (**d**) on PND 16. The scale bar is 50 µm. P27-positive cells per seminiferous tubule among the control and CORT mice (**e**). Values are expressed as the mean ± S.E.M of data from 10 animals per group (10 seminiferous tubules per animal). *p < 0.05 and ***p < 0.001 compared to the control.

In this study, two Sertoli cell markers, GATA-1 and SOX-9, were used to examine the number of Sertoli cells in CORT mice. Immunohistochemistry results revealed a significant decrease in the Sertoli cell number (GATA-1-positive cells and SOX-9-positive cells) per seminiferous tubule in low- and middle-dosed-CORT mice compared with that in control mice on PND 10 (Figs. 5a–e and 6a–e). The number of p27-positive Sertoli cells was significantly higher in low- and middle-dosed-CORT mice than that in control mice (Fig. 7a–e). On PND 16, the number of Sertoli cells per seminiferous tubule was significantly lower in low- and middle-dosed-CORT mice than that in control mice (Figs. 8a–e and 9a–e). The number of p27-positive Sertoli cells was significantly lower in all dosed-CORT mice (Fig. 10a–e).

### Testicular weight, Sertoli cell number, and spermatozoa count in CORT mice on PND 70

**Figure 11:**
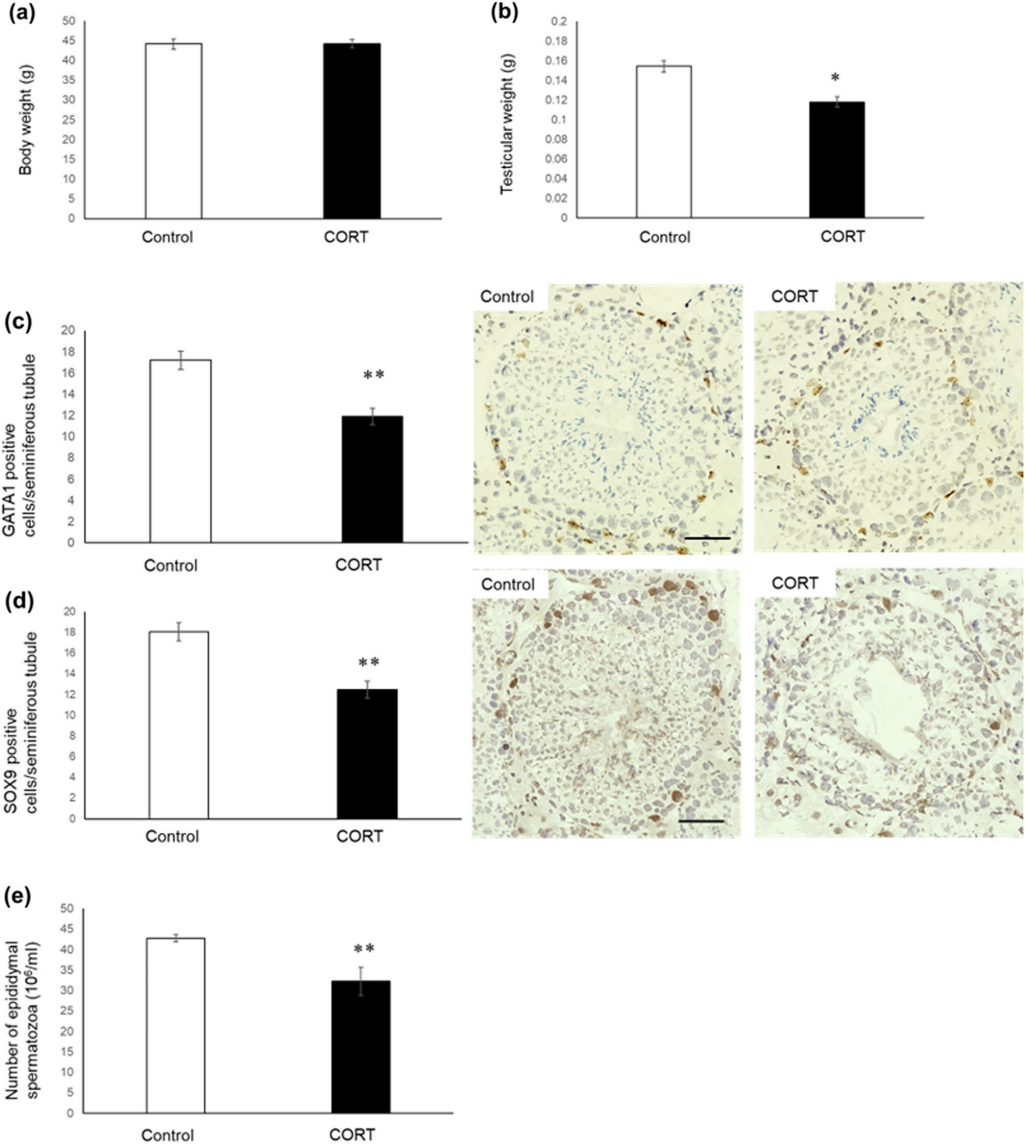
Testicular weight, Sertoli cell number, and spermatozoa count in CORT-administered mice on PND 70. Body weight (**a**), testicular weight (**b**), GATA-1-positive cells per seminiferous tubule (**c**), SOX-9-positive cells per seminiferous tubule (**d**), and spermatozoa count (**e**) on PND 70 were investigated between control and low-dosed-CORT mice. The scale bar is 50 µm. Values are expressed as the mean ± S.E.M of 12 samples per group for body weight, testicular weight, and spermatozoa count. For GATA-1-positive cells, values are expressed as the mean ± S.E.M of data from 10 animals per group (10 seminiferous tubules per animal). *p < 0.05, **p < 0.01 compared to the control.

Our previous study revealed that the 2-h/day NMS mice demonstrated an approximately three-fold increase in serum CORT level compared with control on PND 10, which decreased testicular weight, Sertoli cell number, and spermatozoa count on PND 70^25,26^. In this study, a similar increase in serum CORT levels was observed in low-dosed-CORT mice as compared with those in control on PND 10 (Fig. 1). On PND 70, no difference was observed in BW between control and low-dosed-CORT mice (Fig. 11a). The testicular weight of low-dosed-CORT mice was significantly lower than that of control mice (Fig. 11b). The immunohistochemistry results revealed a significant decrease in the Sertoli cell number in low-dosed-CORT mice (Fig. 11c,d). Also, a significant decrease in spermatozoa count was observed in low-dosed-CORT mice (Fig. 11e).

## Discussion

In this study, neonatal CORT administration at low and middle doses resulted in increased p27-positive Sertoli cell numbers (Fig. 7). P27 is one of the CDK inhibitors involved in the termination of Sertoli cell proliferation^27^. In mice, the expression level of p27 in Sertoli cells has been reported to be modest on PND 10, which then increases on PND 16^36^; meanwhile, Sertoli cell proliferation terminates on approximately PND 15^37,38^. Sertoli cells provide both physical and nutritional assistance to germ cells in the seminiferous tubules. An adequate number of Sertoli cells is required to facilitate spermatogenesis, and the number of Sertoli cells is generally determined during the prepuberty phase. Remarkably, the upregulation of p27 expression decreases the number of Sertoli cells, whereas the attenuation of p27 expression increases the number of Sertoli cells^39^. Perturbation of the endocrine system may alter p27 expression levels in Sertoli cells, thereby altering the number of Sertoli cells^36^. The link between CORT treatment and p27 induction in vitro has gained much attention. Zhu et al. reported that p27 in ductal mammary epithelial cells increased dose-dependently in response to increasing corticosterone administration^40^. Furthermore, Jiang et al. reported that CORT induced p27 expression in mouse mammary hyperplastic epithelial cell lines (TM-10 cells)^32^. On the other hand, our findings clarified that p27 expression was induced in Sertoli cells following CORT exposure in vivo. In addition, immunohistochemistry results revealed that the number of Sertoli cells decreased in low- and middle-dosed-CORT mice on PNDs 10 and 16 (Figs. 5, 6, 8 and 9). The decrease in Sertoli cells is believed to be induced by p27 upregulation. Therefore, in this study, we identified that CORT is one of the endocrine substances that can induce p27 expression and terminate Sertoli cell proliferation in vivo.

In our previous study, we reported that NMS increased the number of p27-positive Sertoli cells and decreased the number of Sertoli cells on PNDs 10 and 16 in response to an increase in serum CORT levels^26^. NMS is a representative model for inducing ELS, and maternal separation results in complex adverse effects, including loss of body temperature and skin-to-skin interaction because of the absence of the maternal mouse, malnutrition, emotional distress, and disturbance of several hormones (such as an increased CORT level), on neonates^25,26^. This study demonstrated that CORT administration increased the p27-positive Sertoli cell number and decreased Sertoli cell number on PNDs 10 and 16 (Figs. 5, 6, 7, 8, 9, 10). These results suggest that CORT induced following maternal separation is one of the triggers for the decreased Sertoli cell number in the testes of NMS mice.

Testosterone and T3 were also reported to regulate p27 expression levels. Buzzard et al. demonstrated that T3 and testosterone treatments increased p27 expression in cultured rat Sertoli cells^28^. Holsberger et al. documented the p27 level in the Sertoli cells of hyperthyroid neonatal mice^36^. In the present study, serum T3 levels were significantly increased on PND 10 in low-dosed-CORT mice but decreased in middle- and high-dosed-CORT mice (Fig. 1c), and testosterone levels were significantly increased in only high-dosed-CORT mice (Fig. 1b); meanwhile, the p27 level was increased in low- and middle-dosed-CORT mice. Therefore, serum testosterone and T3 levels vary under different experimental conditions (low-, middle-, and high-dosed-CORT mice). However, whether these hormones are involved in p27 upregulation and Sertoli cell proliferation following neonatal CORT administration is ambiguous. The different physiological mechanisms for each experimental condition may be involved in the regulation of serum hormone levels. However, it is possible that the administered CORT directly increased p27 expression levels. Several studies have also found that NR3C1, one of the GRs, is expressed in Sertoli cells^33–35^. Additional experiments may be required to determine the precise mechanism by which CORT administration causes p27 upregulation in Sertoli cells. To investigate this mechanism, we plan to treat Sertoli cells isolated from prepubertal mice cells with CORT and the glucocorticoid antagonist RU 486, and analyze the p27 expression level. Remarkably, Jiang et al. found that CORT induced p27 expression and RU 486 prevented p27 induction following CORT treatment in TM-10 cells^32^.

In the present study, an increase in the number of p27-positive Sertoli cells and a decrease in the number of Sertoli cells in low- and middle-dosed-CORT mice, but not in high-dosed-CORT mice were observed on PND 10. The reason behind this finding is still unknown. According to several studies, exposure to stress and chemical compounds causes monotonic and nonmonotonic response patterns^41,42^. In NMS, inverted U-shaped and multiphasic response patterns were found to coexist^25,26^. The administration of CORT in this study is assumed to have an inverted U-shaped response pattern for p27-positive Sertoli cell and Sertoli cell numbers on PND 10. On the other hand, we detected a significant decrease in the number of p27-positive Sertoli cells in high-dosed-CORT mice as well as low- and middle-dosed-CORT mice on PND 16, although no changes in the Sertoli cell number were observed. We cannot explain why the number of p27-positive Sertoli cells was decreased in high-dosed-CORT mice. Some studies reported that exposure to CORT changed DNA methylation levels^43,44^. Exposure to CORT is involved in *Nr3c1* hypermethylation in the midbrain of male mice^45^. Other studies also indicated that ELS caused an increase in the methylation level of the promoter region of *NR3C1* and decreased its expression level^46,47^. Such epigenetic dysregulation may disturb the p27 expression level, as p27 is a target of glucocorticoids and GRs. CDK inhibitors, such as *p27* and *p16*, were also highly methylated in their promoters to silence these genes in several cancers^48^. Unfortunately, no studies have reported on the dysregulation of methylation levels in the promotor region of *NR3C1* and *p27* in Sertoli cells following exposure to CORT. We aim to identify epigenetic dysregulation following the administration of CORT in Sertoli cells in future studies.

A previous study showed that CORT induction in early life stages affected the nervous and endocrine systems^20^. However, information regarding the effects of CORT administration on the male reproductive system during the early life stage is insufficient. In this study, we identified that CORT administration during early life stages has adverse effects on the male reproductive system. We reported an increase in p27-positive Sertoli cell number and a decrease in Sertoli cell number after CORT administration during PND 1–10. We also described the hypothesis for toxicological mechanisms of CORT in testes. In Sertoli cells, there is a possibility that CORT may increase p27 level via GR and decrease Sertoli cell number. Indeed, in addition to p27 upregulation, we considered that several other physiological pathways may be involved in decreasing the Sertoli cell number. For example, CORT treatment causes apoptosis in several tissues and cultured cells^49,50^. Currently, toxicological mechanisms of exposure to CORT in Sertoli cells and testes in early life are unclear. Additional in vivo and in vitro investigations are needed to determine the precise effects and toxicological processes of CORT treatment on Sertoli cells and testes. In addition, it should be noted that exposure to CORT during early life stages reportedly induces a change in GR expression level through epigenetic dysfunction in the nervous system. Such changes in GR expression level have been reported to be associated with life-long alterations in anxiety, fear, and sociability-like behavior^51^. Changes in GR expression levels in Sertoli and germ cells and consequent health effects on the male reproductive system following neonatal CORT administration remain unclear. In addition to the toxicological effects of CORT at prepubertal stages, the present data indicated that increased serum CORT levels in early life stages could decrease testicular weight, Sertoli cell number, and spermatozoa count in the adult stage (Fig. 11). Further studies are required to fully understand the effects of CORT exposure on the male reproductive system during early life stages.

## Methods

### Animals

Ten-week-old ICR male and female mice were purchased from Sankyo-lab. The mice were housed under a 12-h light/dark cycle at a controlled temperature (24–26 °C). Standard chow (F-2, Funabashi Farm Co., Funabashi, Japan) and water were provided ad libitum. Two weeks later, they were mated. After copulation plugs were found, females were separated from male mice. After delivery, CORT administration was performed. All experiments were conducted as per the ethics committee of the animal laboratory at Tokyo Medical University (approval numbers: H31-0050 and R2-0043). All experiments performed followed relevant guidelines and regulations and complied with the ARRIVE guidelines.

### CORT administration

Biagini et al. reported that CORT administration at a dose of 10 mg/kg BW/day in neonates results in adverse effects on the male reproductive system^22^. Referring to their study, we chose a CORT dose to be applied to mice in this study. CORT administration was performed according to our previous study^52^. In brief, the pregnant dams were randomly divided into four groups (3–4 pregnant dams/group). CORT (27840, Sigma-Aldrich, St. Louis, MO, USA) was dissolved in dimethyl sulfoxide (049-07213, Wako Pure Chemical Industries Ltd., Osaka, Japan), and sesame oil (S3547, Sigma–Aldrich, St. Louis, MO, USA). CORT was subcutaneously injected at doses of 0.36, 3.6, and 36 mg/kg BW from PND 1 to 10 (low-, middle-, and high-dosed-CORT mice, respectively). The control mice were injected with dimethyl sulfoxide and sesame oil.

On PNDs 10, 16 and 70, 4–5 male pups were randomly selected from each litter, deeply anesthetized with pentobarbital, and euthanized following terminal cardiocentesis; testis samples were then collected (12–15 male pups/group).

### Histology

The removed testes were immediately fixed with Bouin’s solution and embedded in plastic (Technovit 7100; Kulzer & Co., Wehrheim, Germany), without cutting the organs to avoid artificial damage to the testicular tissues. Sections (5 µm) were obtained at 25–30-µm intervals and stained with Gill’s hematoxylin III and 2% eosin Y for observation under a light microscope (BX-51, Olympus Optical Co., Tokyo, Japan). The diameters of seminiferous tubules, height of the seminiferous epithelia, and relative interstitial areas were measured using the ImageJ 1.51J8 program (National Institutes of Health, USA) (Supplementary Fig. S1).

### Immunohistochemistry

Test samples from control and all-dosed-CORT mice were embedded with Tissue-Tek OCT compound, frozen in liquid nitrogen, and stored at − 80 °C until examination. Embedded testes were dissected with a cryostat at 5 m. The sections were fixed with acetone for 2 min at − 20 °C. For immunohistochemistry, separate sections were incubated with primary antibodies: rabbit anti-p27 polyclonal antibody (ab190851, Abcam; 1:100 dilution) that recognizes CDK inhibitor, rat anti-GATA-1 monoclonal antibody (N6) (sc-265, Santa Cruz Biotechnology, Inc.; 1:100 dilution) or rabbit anti-SOX-9 polyclonal antibody (ab5535, Millipore; 1:2000 dilution), that recognizes a marker of Sertoli cells. Following overnight reaction, sections were washed and incubated with a biotinylated anti-rabbit or rat immunoglobulin IgG (1:200) for 30 min and labeled with avidin-biotinylated horseradish peroxidase (PK-6101, Vectastain ABC Elite Kit, Vector Laboratories, Burlingame, CA, United States) for 30 min at room temperature. Following washing, immunoreactivities were visualized with 3,3′-diaminobenzidine (DAB), and sections were counterstained with Gill’s hematoxylin III. Twenty circular seminiferous tubules were randomly selected for each animal, and immunoreactive cells were counted using the ImageJ 1.51J8 program.

### Enzyme-linked immunosorbent assay (ELISA)

Serum T3, testosterone, and CORT levels were measured using ELISA, as described by Miyaso et al.^26^. Briefly, whole blood (collected at euthanasia by cardiac puncture) was clotted and processed by using standard techniques; the resulting serum samples were collected and stored at − 80 °C until analyses. ELISAs were conducted as per the manufacturer’s protocols [T3043T-100 (CALBIOTECH), 55-TESMS-E01 (ALPCO Diagnostics), ADI-900-097 (Enzo Life Sciences)].

### Spermatozoa count

Spermatozoa count was performed as described by Miyaso et al.^25^. Epididymides were placed in Hanks’ medium and cut open using dissection scissors to retrieve spermatozoa. The resultant suspensions were passed through nylon mesh to eliminate residues from the epididymal tissues. A hemocytometer was used to count spermatozoa using light microscopy.

### Statistics

Statistical analyses comparing the control and experimental mice (low-, middle-, and high dose groups) were conducted using a two-tailed Kruskal–Wallis test with a post hoc Steel test, using EZR version 1.36 (Saitama Medical Center, Jichi Medical University, Saitama, Japan)^53^. A p-value of < 0.05 was considered significant. Where appropriate, values are represented as the mean ± standard error of the mean (S.E.M).

## Supplementary Information

Supplementary Figure S1.

## Data Availability

All data generated or analyzed during this study are included in this published article.
