# Supplementary material for: Neonatal corticosterone administration increases p27-positive Sertoli cell number and decreases Sertoli cell number in the testes of mice at prepuberty
Source: Sci Rep. 2022 Nov 12;12:19402. doi: 10.1038/s41598-022-23695-8 (PMC9653474; doi:10.1038/s41598-022-23695-8)

Neonatal corticosterone administration increases p27-positive Sertoli cell number and decreases Sertoli cell number in the testes of mice at prepuberty

Hidenobu Miyaso[1, 2*], Kaiya Takano[2], Kenta Nagahori[2], Zhong-Lian Li[2], Shinichi Kawata[2], Miyuki Kuramasu[2], Yuki Ogawa[2], Hirotaka Yoshioka[1], Yoshiharu Matsuno[3], Satoshi Yokota[4], and Masahiro Itoh[2]

[1] Department of Anatomy, Faculty of Medicine, School of Medicine, International University of Health and Welfare, 4-3 Kozunomori, Narita, Chiba, 286-8686, Japan.

[2] Department of Anatomy, Tokyo Medical University, 6-1-1 Shinjuku, Shinjuku-ku, Tokyo 160-8402, Japan.

[3] Center for Basic Medical Research, Narita Campus, International University of Health and Welfare, 4-3 Kozunomori, Narita, Chiba, 286-8686, Japan.

[4] Division of Cellular & Molecular Toxicology, Center for Biological Safety & Research, National Institute of Health Sciences, 3-25-26 Tono-machi, Kawasaki-ku, Kawasaki, Kanagawa 210-9501, Japan.

Address correspondence to:

Hidenobu Miyaso, D.Med.Sci.

Department of Anatomy, Faculty of Medicine, School of Medicine, International University of Health and Welfare, 4-3 Kozunomori, Narita, Chiba, 286-8686, Japan.
Tel: +81-476-20-7701
Fax: +81-476-20-7702
Email: h-miyaso@iuhw.ac.jp

Supplemental Figure S1. The diameter of seminiferous tubules and the height of the seminiferous epithelia in testicular tissues.

The diameter of seminiferous tubule (a) and the height of seminiferous epithelia (b) in testicular tissues are marked on the micrographs. The scale bar is 100 μm.

Figure S1.

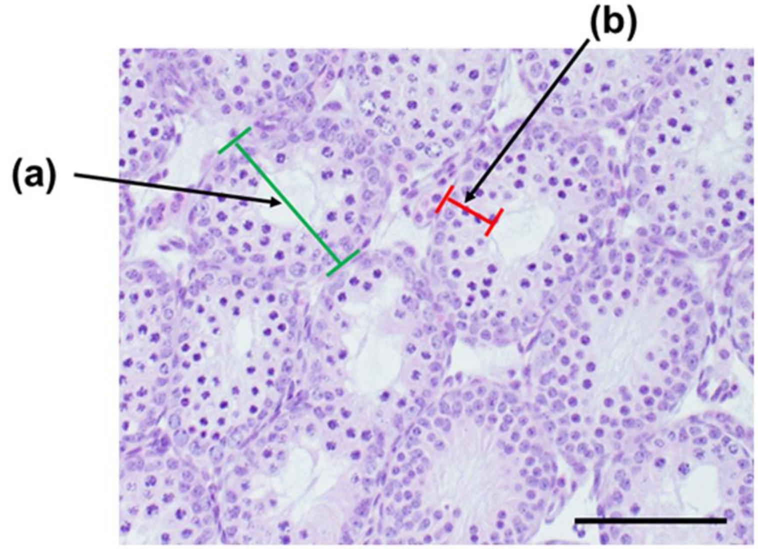

Supplement: Supplementary file 1 — Supplementary Figure S1. [file 41598_2022_23695_MOESM1_ESM.pdf]
